# Oriental Medicine Kyung-Ok-Ko Prevents and Alleviates Dehydroepiandrosterone-Induced Polycystic Ovarian Syndrome in Rats

**DOI:** 10.1371/journal.pone.0087623

**Published:** 2014-02-10

**Authors:** Minhee Jang, Min Jung Lee, Jin Moo Lee, Chun-Sik Bae, Sung-Hoon Kim, Jong Hoon Ryu, Ik-Hyun Cho

**Affiliations:** 1 Department of Convergence Medical Science, Kyung Hee University, Seoul, Republic of Korea; 2 Department of Cancer Preventive Material Development, Kyung Hee University, Seoul, Republic of Korea; 3 Department of Gynecology, College of Korean Medicine, Kyung Hee University, Seoul, Republic of Korea; 4 College of Veterinary Medicine and Animal Medical Institute, Chonnam National University, Gwangju, Republic of Korea; 5 Institute of Korean Medicine, Kyung Hee University, Seoul, Republic of Korea; 6 Department of Oriental Pharmaceutical Science, College of Pharmacy, Kyung Hee University, Seoul, Republic of Korea; 7 Department of Life and Nanopharmaceutical Science, Kyung Hee University, Seoul, Republic of Korea; College of Tropical Agriculture and Human Resources, University of Hawaii, United States of America

## Abstract

Kyung-Ok-Ko (KOK), a traditional herbal prescription composed of *Rehmannia glutinosa* Liboschitz var. *purpurae*, *Lycium chinense*, *Aquillaria agallocha*, *Poria cocos*, *Panax ginseng*, and honey, has been widely used in traditional Oriental medicine as a vitalizing medicine or as the prescription for patients with age-associated disorders such as amnesia and stroke. However, the potential protective value of KOK for the treatment of polycystic ovarian syndrome (PCOS) is largely unknown. We investigated whether pre-administration (daily from 2 hours before PCOS induction) and post-administration (daily after induction of PCOS) of KOK (0.5, 1.0, and 2.0 g/kg/day, p.o.) could have a protective effect in a dehydroepiandrosterone (DHEA, s.c.)-induced PCOS rat model. Pre-administration of KOK significantly decreased the elevated body weight and ovary weight, elevated size and number of follicular cysts, elevated level of serum glucose, and estradiol after DHEA injection. KOK reduced the elevated percentage of CD8 (+) T lymphocytes in lymph nodes, the elevated mRNA expression of CD11b and CD3 in ovaries, and infiltration of macrophages in ovarian tissue with PCOS. KOK diminished the increased mRNA expression of pro-inflammatory cytokines (IL-1β, IL-6, TNF-α), chemokines (IL-8, MCP-1), and iNOS in the ovaries, and increased the reduced mRNA expression of growth factors (EGF, TGF-β) by DHEA injection. Post-administration of KOK also improved the DHEA-induced PCOS-like symptoms, generally similar to those evident from pre-administration of KOK. KOK may effectively prevent and improve DHEA-induced PCOS via anti-inflammatory action, indicating its preventive and therapeutic potential for suppressing PCOS.

## Introduction

Polycystic ovary syndrome (PCOS) is the most common and complex endocrine disorder of young women, estimated to affect 5-10% of women through and beyond their reproductive years [1], [2]. The prevalence of affected individuals and the wide range of related phenotypes are due to environmental and genetic factors [3]. The presence of a polycystic ovarian morphology is a criterion for defining PCOS. The major clinical features of PCOS are chronic oligo- or anovulation, impaired fertility, and metabolic disturbances associated with hyperinsulinaemia, diabetes mellitus, hyperandrogenia, hirsutism, acne, and increased incidence of endometrial cancer [1], [3]–[5]. PCOS is strongly associated with abdominal obesity and hyperlipidaemia; in one study, 43% of women with PCOS had a higher risk of developing hyperinsulinemia, insulin resistance, and type 2 diabetes [1], [3], [4]. Women with PCOS also tend to have a higher ratio of luteinizing hormone to follicle-stimulating hormone, which disrupts the ovarian follicular development and anovulation [5]. Furthermore, the disruption in the level of estrogen that can occur along with severe oligomenorrhea and amenorrhea can also produce endometrial hyperplasia and cervical cancer [6].

Many patients with PCOS require prolonged periods of treatment, and the primary goal of treatment is normalization of androgen levels and restoration of reproductive function. Metabolic disturbances that appear in women with PCOS may increase the risk for cardiovascular disease and may exacerbate many of the PCOS-like symptoms. Thus, metabolic disturbances are considered as an important target for therapy. The implication of obesity and insulin resistance in PCOS strongly suggests that reduction of these risk factors should be a central focus for a long-term treatment of women with PCOS [7]. Treatment can involve many therapies hormone therapy, ovulation induction, surgery. A pharmacological approach using clomiphene citrate to curb the production of estrogen is the first-line treatment to enhance the induction of ovulation in women with PCOS [8].

Recent studies have investigated the role of metformin (*N,N′-dimethyl-biguanide*) as insulin-sensitizing agents. The use of metformin is becoming increasingly accepted and widespread. Despite this increasing clinical use, the details of the mechanism of action of metformin are incomplete [9], [10]. Furthermore, it can cause multiple follicular development with the risk of ovarian hyperstimulation and multiple pregnancy, and can increase the risk of ectopic pregnancy and congenital malformations, such as neural tube defects, producing an unsatisfactory treatment outcome [11]. Complementary and alternative therapies including acupuncture, Chinese herbal medicine, and dietary supplements can alleviate PCOS or reduce the side effects of these chemicals for PCOS [12]. Unfortunately, there is minimal evidence that complimentary and alternative therapy is safe and efficacious. Therefore, new treatment strategies including complimentary and alternative therapy need to be evaluated to alleviate PCOS, regulate hormones, and improve quality of life in PCOS patients.

Kyung-Ok-Ko (KOK; Qiong-yu-gao in Chinese; Kei-gyoku-kou in Japanese) is a traditional oriental prescription that consists of a decoction of six ingredients: *Rehmannia glutinosa Liboschitz* var. *purpurae* Makino (Scrophulariaceae), *Lycium chinense* Miller (Solanaceae), *Aquillaria agallocha* Roxburgh (Thymelaeaceae), *Poria cocos* Wolf (Polyporaceae), *Panax ginseng* C.A. Meyer (Araliaceae), and honey. In East Asia, KOK is administered as vitalizing medicine to healthy people or with medicinal intent to patients with various age-related symptoms, such as lack of vigor and immunity, emaciation/weakness, stroke, amnesia, and dementia [13]. Benefits attributed to KOK include anti-oxidation [14], anti-tyrosinase [15], and anti-cancer activity [16]. Recently, we reported that KOK improves scopolamine-induced cognitive impairments in mice [17]. It seems that the therapeutic mechanisms and efficacies of KOK are likely varied and desirable. Yet, their effectiveness has not been clearly elaborated. Various resent studies have sought to develop anti-PCOS drugs from natural products and traditional prescriptions [18], [19]. But, whether KOK has protective or ameliorative effects against PCOS is unknown. We investigated whether KOK is a beneficial treatment for PCOS using a dehydroepiandrosterone (DHEA)-induced PCOS rat model. The results indicate that KOK has preventive and therapeutic benefits in the treatment of PCOS.

## Materials and Methods

### Animals

Nine Sprague Dawley dams (Narabiotec, Seoul, Korea), each with eight to nine female pubs, were kept at a constant temperature of 23±3°C with a 12-hours light-dark cycle (light on 08:00 to 20:00), and fed food and water *ad libitum*. The animals were allowed to habituate to the housing facilities for 1 week before the experiments. All experimental procedures were reviewed and approved by the Institutional Animal Care and Use Committee (IACUC) of Kyung Hee University [KHUASP (SE)-12-030].

### KOK preparation

KOK (Lot No. SQ12, 100 g) was obtained from Kwang Dong Pharmaceutical Company (Pyongtaek, Korea). The preparation was manufactured as previously described [20]. Briefly, juice from the root of *Rehmannia glutinosa* Liboschitz var. *purpurae* Makino (32.0 g), powder of dried fruit of *Lycium chinense* Miller (0.9 g), powder of resin of *Aquillaria agallocha* Roxburgh (0.1 g), powder of cortex of *Poria cocos* Wolf (8.0 g), powder of dried root of *Panax ginseng* C.A. Meyer (2.8 g), honey (38.5 g of native acacia honey), and simple syrup (17.7 g of 85% sucrose solution) were mixed and heated in a water bath at 80°C for 72 hours. The resulting viscous extract was stored at 4°C in a sealed jar and used without further preparation. The shelf life of KOK is 3 year at a constant temperature of 25±2°C and relative humidity of 60±5% according to manufacture data. A long-term storage test manufacturer conducted to determined/confirm stability of KOK did not reveal any significant change in formal analysis, quality control criteria of raw ingredients, loss of dry weight (28.5–34.5%), ethanol dried extract (4.9–5.7 g/10 g), water dried extract (5.0–5.8 g/10 g), mineral (under 3.0%), acid-insoluble minerals (under 1.5%), heavy metals (under 30 ppm), and maximum residue limit (benzene hexachloride, under 5 ppm; dichloro-diphenyl-trichloroethane, under 3 ppm) at 0, 3, 6, 9, 12, 18, 24, and 36 months after manufacture.

KOK was standardized with 5-hydroxymethylfurfural (9.4%) for consistency of quality by the Kwang Dong Pharmaceutical Company (Materials and Methods S1). Quality control criteria of the major raw ingredients were accomplished using column chromatography at this company according to the method outlined in the Korea Food Code [21]. One or two spots from samples of *Rehmannia glutinosa* Liboschitz var. *purpurae*, *Poria cocos*, and *Panax ginseng* were same in reference value and color compared with each standard solution.

### PCOS induction and KOK treatment

**Figure 1:**
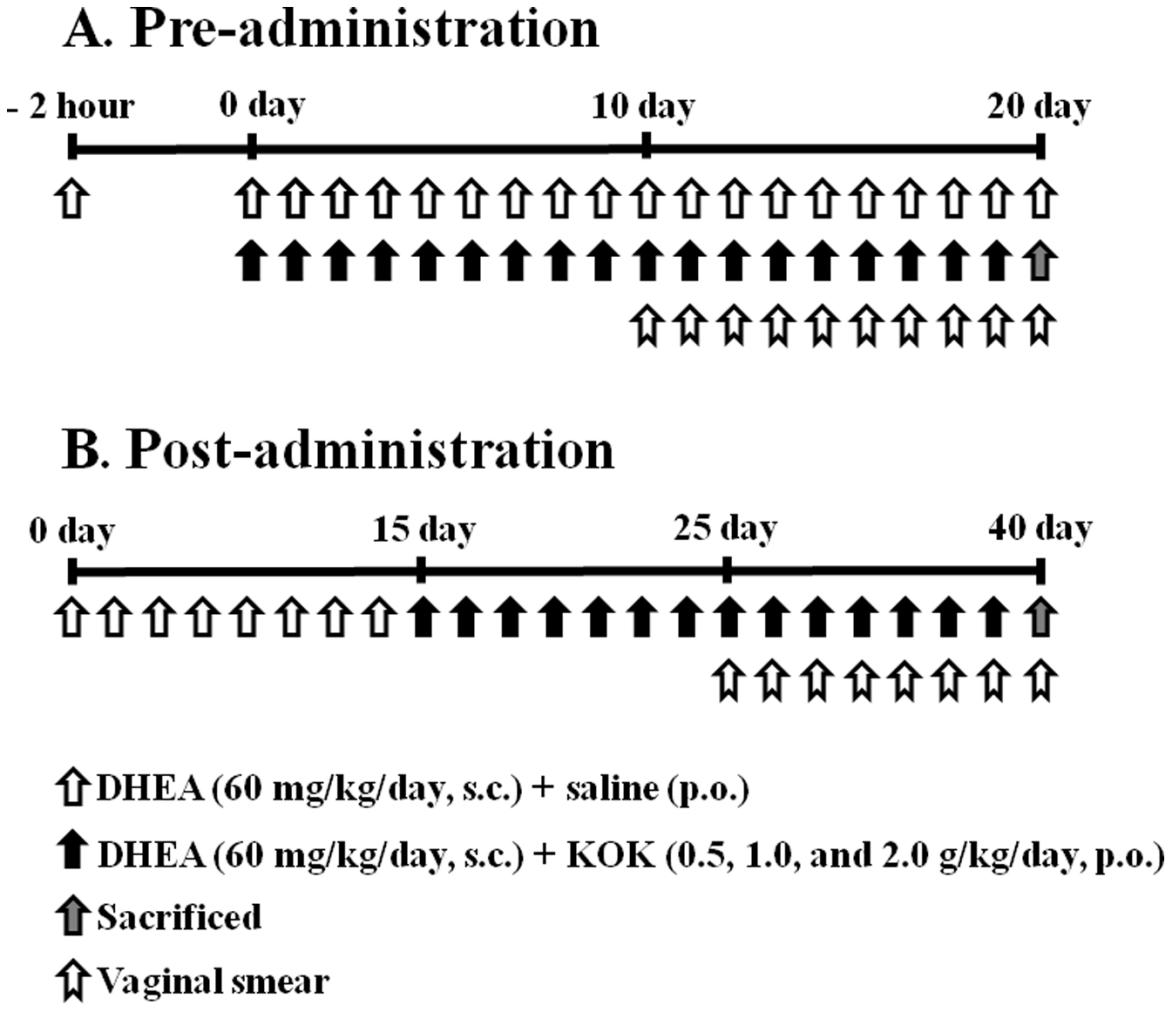
Experimental protocols for PCOS model and KOK treatment. (**A**) Pre-administration of KOK (daily from 2 h before first DHEA injection). (**B**) Post-administration of KOK (daily from 15 days after PCOS induction by DHEA injection). DHEA and DHEA+KOK groups were subcutaneously injected with 60 mg/kg/day of DHEA for 20 days (A) or 40 days (B) and received daily intraperitoneal KOK from day 0 (A) or 15 (B) to day 20 (A) or 40 (B). The KOK group was treated with KOK alone. Vaginal smear was examined daily from 10 day (A) or 15 day (B) after DHEA injection.

Female, 23-day-old, prepuberal rats were allocated into two experimental groups for pre-administration and post-administration of KOK, and each group was subdivided into six subgroups (n = 9 in each subgroup): (Fig. 1). Subgroup 1 was the normal control group [saline, subcutaneous (s.c.) + saline, per os (p.o.)]. Subgroup 2 was the DHEA treatment group [DHEA, 60 mg/kg body weight/day, s.c.+ saline, p.o.]. Subgroups 3, 4, and 5 comprised the DHEA+KOK treatment groups [DHEA, 60 mg/kg body weight/day, s.c.; KOK, 0.5 (subgroup 3), 1.0 (subgroup 4), or 2.0 (subgroup 5) g/kg body weight/day, p.o.]. Subgroup 6 was the KOK treatment group (saline, s.c.+2.0 g/kg body weight/day of KOK, p.o.). The PCOS rat model involved injection of DHEA [22]. Briefly, rats in the DHEA group received a daily injection of DHEA dissolved in 0.2 ml sesame oil beginning at 23-days-of-age. To test the preventive effect of pre-administration of KOK in PCOS, KOK was administrated orally daily for 20 consecutive days 2 hours before the first injection of DHEA. Rats in the normal or KOK groups were administrated saline or KOK without DHEA injection. The dose of KOK was determined by a preliminary experiment for this study, by our previous report [20], by traditional prescription [13], and by well-known formula [23]. To evaluate the therapeutic effect of post-administration with KOK on PCOS, 15 days after the first DHEA injection the rats were randomly subdivided into the aforementioned six subgroups. Rats in each subgroup received DHEA, DHEA+KOK, or KOK as previously detailed for 40 days. The experiment was performed more three times using the same protocol.

### Body weight and vaginal smears

Body weight was determined daily during the experimental period, before treatment with DHEA, KOK, or saline. The stage of estrous cyclicity was also determined by microscopic analysis of the predominant cell type in vaginal smears obtained daily beginning on day 10 (33 days after birth) in KOK-pre-administrated rats and on day 25 (48 days after birth) in rats post-administrated KOK after DHEA injection. Assessments continued to the end of the experiment as previous described [24].

### Blood sampling and glucose, insulin, estradiol, and progesterone determination

At 20 days (pre-administration of KOK) and 40 days (post-administration of KOK) after DHEA injection, rats were fasted for 6 hours (8:00 a.m. to 2:00 p.m.) [25] prior to euthanization by an intraperitoneal overdose of sodium pentobarbital. Heart blood was collected in anticoagulant vials to assess glucose, insulin, estradiol, and progesterone concentrations. Blood was centrifuged at 3,000 rpm for 10 minutes. Serum samples were stored at −80°C. Serum glucose concentrations were determined using Pureauto S GLU reagent (Sekisui, Osaka, Japan) by standard enzymatic methods on a 7600-210 autoanalyser (Hitachi, Tokyo, Japan). Serum insulin concentration was measured using an enzyme-linked-immunosorbent serologic assay (ELISA) with an Ultrasensitive Rat Insulin ELISA Kit (Mercodia, Uppsala, Sweden) according to the manufacturer's instructions. These results were used to calculate the Homeostasis Model of Assessment of Insulin Resistance (HOMA-IR), which was determined using the formula [fasting insulin (pM)×fasting glucose (mM)) ÷ 405] [26] as an index of insulin resistance. Serum estradiol and progesterone levels were measured by an electrochemiluminescence immunoassay (Roche Diagnostics GmbH, Mannheim, Germany) as described before [27].

### Histological examination

Twenty and 40 days after the DHEA injection, after taking blood from heart, the ovaries were removed and fixed at 4°C overnight in fresh 4% paraformaldehyde in 0.1 M phosphate buffer (pH 7.4), washed in tap water, embedded in paraffin, and cut into 5 μm-thick sections. The sections were deparaffinized with xylene, rehydrated with graded ethanol, stained with hematoxylin-eosin (H&E) dye, and cover-slipped with Permount. Images of stained sections were visualized and captured using a DP70 digital light microscope system (Olympus, Tokyo, Japan). The number of cysts was counted microscopically in low/high-power fields in a section of the midline from ovaries of each group.

### Immunohistological examination

Immunohistochemical evaluation was performed as previously described [28]. Briefly, ovary sections were incubated for 30 min with 3% hydrogen peroxide in 0.1 M phosphate buffered saline (PBS, pH 7.4) and then blocked with a solution containing 5% normal goat/or horse serum, 2% bovine serum albumin, 2% fetal bovine serum (FBS), and 0.1% Triton X-100 for 2 hours at room temperature. The sections were incubated overnight at 4°C with either mouse anti-rat CD8a (OX-8) (1∶1,000; BD Biosciences, Franklin Lakes, NJ, USA), or rabbit anti-ionized calcium binding adapter molecule (Iba)-1 (1∶2,000; Wako Pure Chemical, Osaka, Japan). Sections were washed in PBS and incubated with biotinylated mouse/rabbit IgG antibody (1∶200; Vector Laboratories, Burlingame, CA, USA) and with avidin-biotinylated horseradish peroxidase (1∶200; Vector Laboratories) for 1 hour at room temperature. Sections were visualized with 3,3′-diaminobenzidine and cover-slipped with Permount.

### RNA extraction and real time-polymerase chain reaction (PCR)

To examine level of induction and recover of PCOS, the mRNA levels of pro-inflammatory cytokines interleukin (IL)-1β, IL-6, and tumor necrosis factor-alpha (TNF-α), chemokines IL-8 and monocyte chemoattractant protein-1 (MCP-1), growth factors epidermal growth factor (EGF) and transforming growth factor-beta (TGF-β), and inducible nitric oxide synthase (iNOS) were assessed 20 days and 40 days after the DHEA injection and KOK administration. Rats (n = 6 per group) were anesthetized and the ovaries were removed and deep-frozen. Real-time PCR was performed using SYBR Green PCR Master Mix (Applied Biosystems, Franklin Lakes, NJ, USA) as previously described [28]. Briefly, reactions were performed in duplicate in a total volume of 10 μl, each containing 10 pM primer, 4 μl cDNA, and 5 μl SYBR Green PCR Master Mix. The mRNA levels of each target gene were normalized to that of glyceraldehyde 3-phosphate dehydrogenase (GAPDH) mRNA. Fold-induction was calculated using the 2−ΔΔCT method as previously described [29]. Real-time-PCR experiments were performed at least three times, and the mean ± SEM values are presented unless otherwise noted. The oligonucleotide primers are summarized in Table 1.

**Table 1:** PCR primer sequence for PCR analysis.

Gene	Reverse Sequence (5′→3′)	Reverse Sequence (5′→3′)
**CD11b**	GGG ATC CGT AAA GTA GTA GAG AA	AAA GGA GCT SST ACT TCC TGT CT
**CD3**	GAT CCC AAA CTC TCT ATA GCT A	CTT TCA TGC CAA TCT CAC TGT G
**IL-1β**	GAC CTG TTC TTT GAG GCT GAC	TTC ATC TCG AAG CCT GCA GTG
**IL-6**	CAA GAG ACT TCC AGC CAG TTG C	TGG CCG AGT AGA CCT CAT AGT GAC C
**TNF-α**	TGA TCG GTC CCA ACA AGG A	TGC TTG GTG GTT TGC TAC GA
**IL-8**	ACG CTG GCT TCT GAC AAC ACT AGT	CTT CTC TGT CCT GAG ACG AGA AGG
**MCP-1**	GGC CTG TTG TTC ACA GTT GCT	ACA CCT GCT GCT GGT GAT TCT
**EGF**	TGC CTT GCC CTG ACT CTA C	AGC CAA TGA CAC AGT TGC AC
**TGF-β**	CTT CAG CTC CAC AGA GAA GAA CTG C	CAT GTT GGA CAA CTG CTC C
**iNOS**	TCT GTG CCT TTG CTC ATG ACA	TGC TTC GAA CAT CGA ACG TC
**Fas**	GCT GTC CTG CCT CTG GTG C	AGG CGA TTT CTG GGA CTT TGT
**GAPDH**	AGG TCA TCC CAG AGC TGA ACG	CAC CCT GTT GCT GTA GCC GTA T

### Flow cytometry

For flow cytometry analysis of CD4 (+) and CD8 (+) lymphocytes, at 20 days and 40 days after DHEA injection, rats (n = 6 per group) were anesthetized, lymph nodes were carefully dissected, and a single-cell suspension was prepared and fixed with 2% paraformaldehyde. Cells were washed with 1% FBS in PBS, incubated with mouse anti-rat CD32 (BD Bioscience) for 10 minutes to block the Fc receptor and washed twice with 1% FBS in PBS. Cells were incubated with APC-conjugated mouse anti-rat CD4 (OX-35; BD Bioscience) and PE-conjugated mouse anti-rat CD8a antibodies (OX-8; BD Bioscience) for 30 minutes at 4°C. The cells were washed twice with 2% FBS in PBS and used for flow cytometry. Data were collected on a FACSCalibur flow cytometer (BD Biosciences) and analyzed using CellQuest Pro software (BD Biosciences).

### Toxicity analysis of KOK

To investigate whether KOK has a toxic effect for long-term use in rats, KOK (2.0 and 4.0 g/kg/day) was orally administrated to normal female prepuberal rats (23-days-old) for 22 days. Body weight was measured and serum was obtained at 24 hours after the last administration of KOK. Serum level of alanine aminotranferease (ALT), aspartate aminotransferase (AST), and lactate dehydrogenase (LDH) was measured using an enzymatic or ultraviolet assay with ALT, AST, or LDH detection kit (Roche, Basel, Switzerland) according to the manufacturer's instructions under cobas 8000 modular analyzer (Roche, Basel, Swetzerland). General structure, apoptotic cell death, and proinflammatory cytokines were evaluated by H&E staining, TUNEL staining, immunohistochemistry, and Western blot analysis for capase-3, or real time PCR analysis for TNF-α and fatty acid synthase (FAS), according to pre-described protocols and manufacturer's instructions (ApopTag peroxidase in situ Apoptosis Detection Kit, S7100, Millipore, USA).

### Statistical analysis

Results are expressed as mean ± SEM. The statistical significance of differences between the values was determined using ANOVA with a Fisher's post-hoc test. A statistical difference was accepted at the 5% level unless indicated otherwise.

## Results

### Effect of KOK on body weight of DHEA-induced PCOS rats

**Figure 2:**
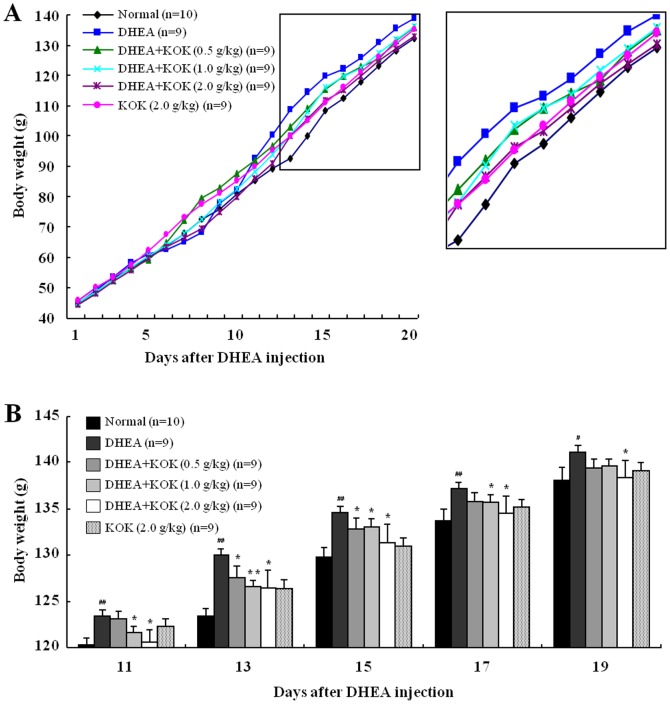
Effect of post-administration of KOK in body weight gain in rats with PCOS. (**A**) Growth curves for the period of the treatment for normal, DHEA, DHEA + KOK, and KOK groups. Values represent the mean. (**B**) Growth graphs for 11–19 days after DHEA injection. Values represent the mean ± SEM. ^#^p<0.05, ^##^p<0.01 normal vs. DHEA group.*p<0.05, **p<0.01 DHEA vs. DHEA + KOK group. ANOVA with a Fisher's post-hoc test.

As a preliminary study to determine the effective dose of KOK on the DHEA-induced PCOS rat model, we tested the effect of KOK in four doses (0.25, 0.5, 1.0, and 2.0 g/kg body weight/day, p.o.). However, KOK 0.25 g/kg/day did not show effect for PCOS model (data not shown). These doses (0.25–2.0 g/kg/day) are similar to recommended doses (5–10 g/adult/day) by Kwang Dong Pharmacological Company as a vitalizing tonic for healthy people. It would seem that effective dosage dependant on animal species and disease type. For future clinical study for PCOS patients, KOK dose has to considered more critically based on various data. Given that metabolic disturbance is one of the major clinical features of PCOS [1], [3], [4], we first investigated whether DHEA-treatment could influence increased body weight in rats with PCOS. A significant increase in body weight was evident from 11 days after DHEA-injection compared to the age-matched normal rats. However, the increased body weight was partially decreased in rats administered DHEA+KOK (Figs. 2A and 2B). Injection of KOK (2.0 g/kg body weight/day) alone did not affect body weight compared to normal rats. The results indicated that pre-administration with KOK could reduce the clinical symptoms including body weight loss of DHEA-induced PCOS in the rat model.

### Effect of KOK on ovarian weight and ovarian morphology

**Figure 3:**
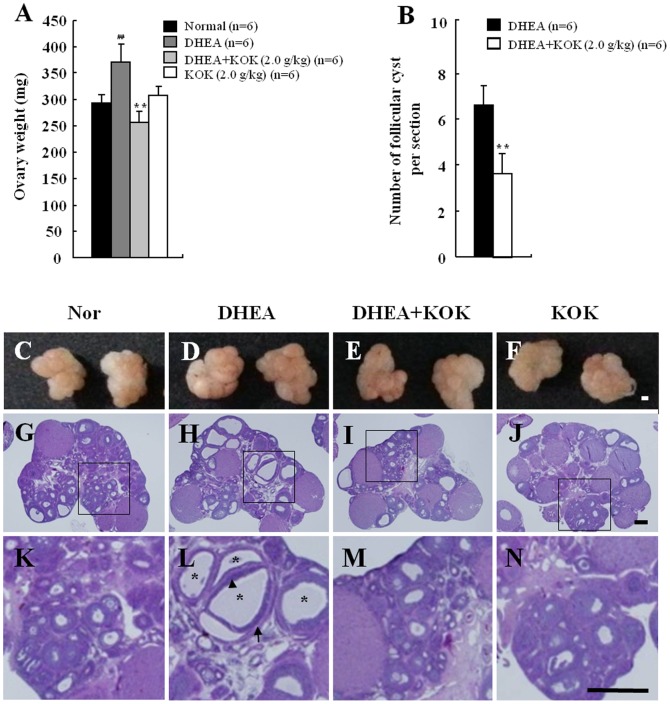
Effect of KOK on ovarian weight and ovarian morphology. (**A**) Ovarian weight. (**B**) Number of follicular cysts. The rectangles indicate the area magnified in photos shown in (C–N). (**C–N**) Photos and photomicrographs of ovaryies from normal (C, G, and K), DHEA (D, H, and L), DHEA + KOK (E, I, and M) and KOK group (F, J, and N). Pre-administration of KOK inhibited the formation of follicular cysts (asterisks) induced by DHEA. Arrow, theca cell layer. Arrow head, granulosa cell layer. Scale bar  = 50 μm.

Since the weight of ovaries from PCOS rats can be changed [3], we examined ovarian weight from each group. The ovarian weights in the DHEA group were higher (by 26.7%) than that in normal group. There was a significant decrease in the DHEA+KOK group (by 30.8%) compared with the DHEA group (Fig. 3A). Injection of KOK alone did not increase or decrease ovary weight compared to normal rats. As the formation of ovarian/follicular cysts mirrors pivotal clinical features of PCOS in humans [3], we investigated whether KOK changed the appearance and histological structures of ovaries. The representative findings of ovaries from each group are shown in Figs. 3B–3N. The ovaries in the normal and KOK groups were normal in appearance with an outward shape, a central medulla consisting of a fibromuscular stroma and a large number of blood vessels, and a peripheral cortex containing large numbers of follicles and large corpora lutea in different stages of growth and regression (Figs. 3C, 3F, 3G, 3J, 3K, and 3N). However, ovaries from rats in the DHEA group exhibited swelled appearance and multiple cystic follicles (Figs. 3D, 3H, and 3L), which were consistent with fully developed PCOS [19], [26], [30]. Ovaries with PCOS in the DHEA group consisted of multiple dilated follicular cysts, atretic follicles, abundant cortical stroma, and well-developed theca interna. Interestingly, the size and number of follicular cysts in the ovaries was decreased in the DHEA+KOK group (Figs. 3B, 3E, 3I, and 3M). The number of follicular cyst in the ovaries in the DHEA group was 6.2±0.7, however the number of cyst in the DHEA+KOK group were 3.4±0.7 (Fig. 3B). These results indicated that the pre-administration with KOK inhibited the development of follicular cysts by DHEA.

### Effect of KOK on metabolic changes after DHEA androgenisation

Given that DHEA-treatment mirrors metabolic changes seen in women with PCOS, we next evaluated whether KOK could regulate the DHEA-mediated increased levels of serum insulin, glucose, and HOMA-IR (Table 2). These alterations resulted in higher values for HOMA-IR in androgenised animals, indicating increased insulin resistance compared to the normal group. In the present study, the elevated level of serum glucose was significantly down-regulated by KOK-pre-administration (Table 2). However, the level of serum insulin and HOMA-IR were not regulated by DHEA treatment or KOK-pre-administration (Table 2). This result suggested that KOK was not associated with insulin resistance in PCOS rats.

**Table 2:** Effect of KOK in fasting glucose, insulin, estradiol, and progesterone levels in rats with PCOS.

	Insulin (ng/ml)	Glucose (mg/dL)	HOMA-IR	Estradiol (E2) (pg/ml)	Progesteron (ng/ml)
**Normal (n = 6)**	0.31±0.04	131.8±3.1	2.4±0.3	5.0±0.0	48.8±25.5
**DHEA (n = 6)**	0.25±0.03	142.2±5.0^#^	2.1±0.3	110.7±53.0^##^	177.9±40.6^##^
**DHEA+KOK (2.0 g/kg) (n = 6)**	0.28±0.02	131.2±7.0^*^	2.1±0.3	22.1±5.3^**^	128.7±22.7
**KOK (2.0 g/kg) (n = 6)**	0.22±0.02	133.3±3.8	2.0±0.2	5.5±0.3	76.8±20.5

Pre-administration of KOK decreased the fasting levels of serum glucose and the level of estradiol except insulin and HOMA-IR. Values represent the mean ± SEM. ^#^p<0.05, ^##^p<0.01 normal vs. DHEA group.*p<0.05, **p<0.01 DHEA vs. DHEA + KOK group, ANOVA with a Fisher's post-hoc test.

### Effect of KOK in ovarian steroidogenic function after DHEA treatment

**Figure 4:**
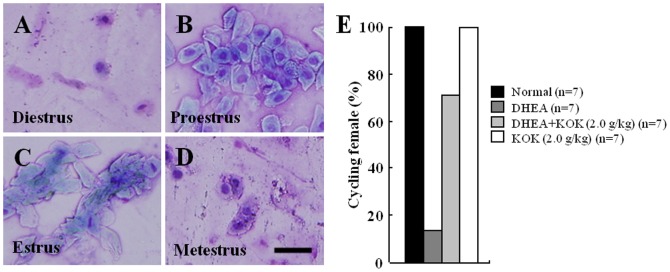
Effect of KOK in estrous cycle in rats with PCOS. (**A–D**) Representative photomicrographs of the vaginal smears stained for Gimsa staining show examples of diestrous (A), proestrous (B), estrous (C), and metaestrous stages (D). (**E**) In the representative graph of pre-administration with KOK, estrous cycle restore was observed by means of the % of females exhibiting complete estrous cycles.

PCOS diagnosis in women depends not only on the formation of ovarian cysts as apparent by ultrasonographically, but also in the presence of oligo- or anovulation and hyperandrogenism [1], [3], [4]. To confirm the presence of these clinical features in DHEA-induced PCOS rats, we analyzed the serum levels of estradiol and progesterone as a marker for steroidogenic function. The serum level of estradiol was significantly increased in the DHEA group (by 22-fold). However, the elevated level of estradiol was abrogated in the DHEA+KOK group (by 80.0%) (Table 2). Although no statistically significant difference was detected, the increased level of serum progesterone in the DHEA group (by 3.6-fold) was slightly decreased in the DHEA+KOK group (by 27.6%) (Table 2). KOK did not affect the serum level of both hormones. Since the regular estrous cycle is a main index of the normal ovarian function in humans, to exam whether KOK or DHEA could affect ovarian function in rats, we confirmed the stage of estrous cycle by vaginal smear (Figs. 4A–4D) with an independent experimental set. All rats in the normal and KOK groups exhibited regular cycling activity at 10–20-days-of-age after starting the experiment (33–43 days after birth). However, the population of rats with estrous stages was reduced to 14.3% in the DHEA group, with recovery to 71.4% in the DHEA+KOK group (Fig. 4E). Taken together, these results indicated the ability of pre-administration with KOK to restore ovarian function by improving the absence of ovarian cycles together with the formation of ovarian cysts.

### Effect of KOK pre-administration in T lymphocyte expression in lymph nodes

**Figure 5:**
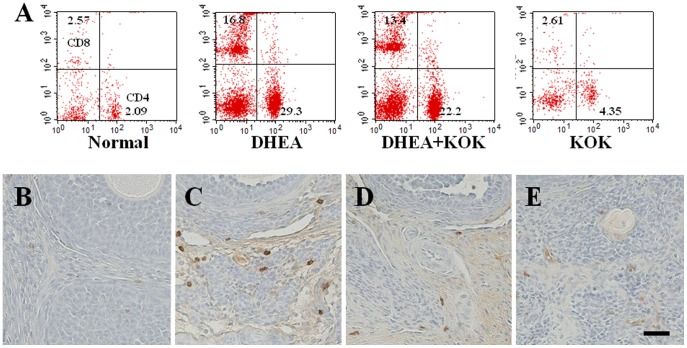
Effect of KOK pre-administration in T lymphocyte expression in lymph nodes and ovaries. (**A**) Lymph nodes were dissected from normal, DHEA treated, DHEA + KOK treated, and KOK alone treated rats (n = 6 per group) at 22 days after DHEA injection to investigate the degree of infiltration of T cell with flow cytometry. Tissues were dissociated, and cells were incubated with APC-conjugated mouse anti-rat CD4 and PE-conjugated mouse anti-rat CD8a antibodies. Representative dot plots from flow cytometric analysis of CD4 (+) and CD8 (+) are displayed. Values (mean ± SEM) display the percentage of CD4 (+) T lymphocytes and CD8 (+) T lymphocytes among lymphocytes. ^#^p<0.01 normal vs. DHEA group. *p<0.05 DHEA vs. DHEA + KOK group. ANOVA with a Fisher's post-hoc test. (**B–E**) Ovarian sections were immunostained with mouse anti-rat CD8a antibody. Representative photomicrographs show that the expression of CD8 (+) T lymphocytes was decreased in DHEA+KOK group (C and D).

A previous study reported increased population and activation of T lymphocytes in lymph nodes in a DHEA-induced PCOS mouse model [22], [31]. To presently study whether pre-administration of KOK affected the frequency and activation status of T lymphocytes, we analyzed CD4 (+) (or helpers) and CD8 (+) (cytotoxic/suppressors) T lymphocytes in the lymph node by flow cytometry (Fig. 5). Lymph nodes of rats in the normal and KOK groups showed equivalent percentages of CD4 (+) and CD8 (+) T lymphocytes (Fig. 5A). However, the percentages of CD4 (+) (29.3±2.6%) and CD8 (+) T lymphocytes (16.8±1.1%) were significantly increased in the DHEA group when compared with normal group. The increased percentage of CD8 (+) T lymphocytes was reduced in the DHEA+KOK group (by 20.9% compared with the DHEA group) (Fig. 5A). Although no statistically significant difference was found, the percentage of CD4 (+) T lymphocytes was slightly decreased by 25% compared with the DHEA group (Fig. 5A). KOK did not affect the population of T lymphocytes in lymph node (Fig. 5A).

T lymphocytes can be recruited to the site of inflammation by ICAM-1 and VCAM-1 [26]. Based on the above effects of KOK on T lymphocytes, we further investigated the presence of CD8 (+) T lymphocytes in the ovarian tissue by immunohistochemistry. Ovarian sections from the normal and DHEA groups harbored CD8 (+) T lymphocytes at very low frequency (Fig. 5). CD8 (+) cells were localized in the theca cell layer of follicles in the stromal tissue and, rarely, in the granulosa cell layer and antrum of a few developing follicles (Figs. 5B–5E). Ovaries from the DHEA group displayed CD8 (+) cells in the granulosa cell layer and antrum of all the cysts. However, in ovaries from the DHEA+KOK group, reduced distribution of CD8 (+) cells (by 21.4%) was evident (Figs. 5B–5E). The results indicated that pre-administration of KOK could down-regulate activation and recruitment of T lymphocytes to polycystic ovaries.

### Effect of KOK pre-administration in macrophage infiltration

**Figure 6:**
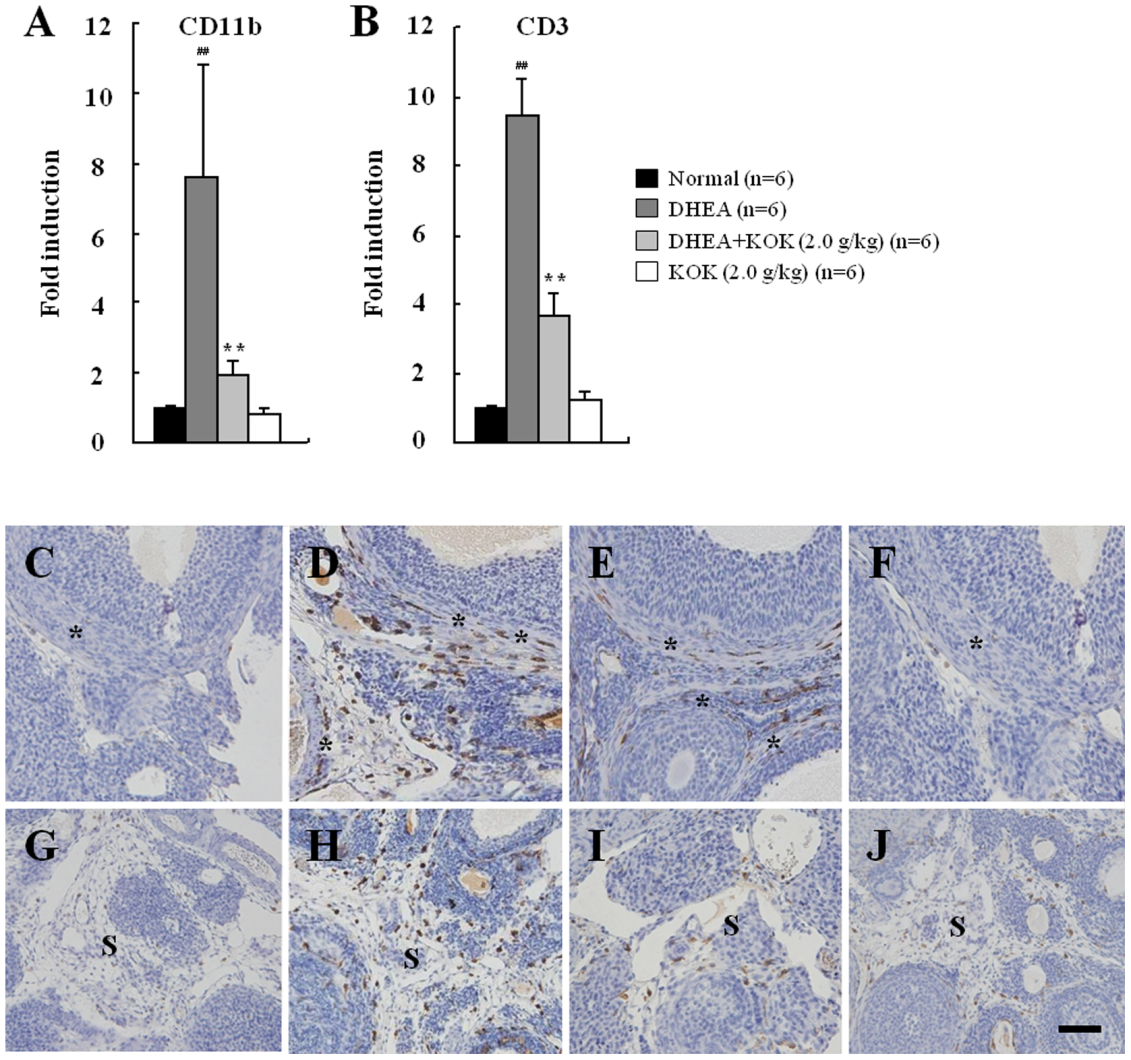
Effect of KOK pre-administration in macrophage infiltration. (**A and B**) Ovarian mRNA expression of CD11b (A) and CD3 (B). Pre-administration of KOK inhibited the elevated mRNA expression of CD11b and CD3 by DHEA. Values represent the mean ± SEM. ^##^p<0.01 normal vs. DHEA group. **p<0.01 DHEA vs. DHEA + KOK group, ANOVA with a Fisher's post-hoc test. (**C–J**) Photomicrographs of ovarian tissue specimens representative for normal (C and G), DHEA (D and H), DHEA + KOK (E and I), and KOK groups (F and J), immunolabeled with Iba-1 antiserum. Ovarian sections from DHEA + KOK group revealed low expression of Iba-1 in the theca cell layer (C–F) of follicles and stroma (G–J) compared with that of DHEA group. In ovaries of normal and KOK group, expression of this marker was rarely observed in theca cell layer of follicles and stroma (C, F, G, and J). Immunohistochemical detection was revealed with DAB, thus, Iba-1 (+) cells appear in brown. Tissue was counterstained with haematoxylin. Asterisks, theca cell layer. S, stromal tissue. Scale bar  = 50 μm.

Macrophages are the most abundant immune cell population in the ovary [32]. Appropriately, we investigated the effect of KOK on infiltration of macrophages in ovarian tissue with PCOS by real time PCR. The mRNA expresstion of CD11b and CD3 increased in ovaries from the DHEA group compared with that of the control group. The up-regulated mRNA expression of CD11b (by 9.4-fold) and CD3 (by 7.6-fold) was significantly down-regulated by 60.9% and 74.3%, respectively, in ovaries from DHEA+KOK group (Figs. 6A and 6B). KOK itself did not regulate the mRNA expression of CD11b and CD3. We also investigated the distribution of macrophages in ovarian tissue by immunohistochemical analysis with Iba-1 anti-serum, as a marker of macrophage. In ovarian tissue in the DHEA group, the number of Iba-1 (+) macrophages was increased in the theca cell layer of follicles, in the stromal tissue (Figs. 6D and 6H). However, the increased Iba-1 immunoreactivity decreased in the DHEA+KOK group (Figs. 6E and 6I). The results suggest that pre-administration of KOK can inhibit the infiltration and activation of macrophage to the site of inflammation of ovarian tissue with PCOS.

### Effect of KOK on the expression of inflammatory mediators

PCOS in humans has been linked to chronic inflammation [33]. The major pathogenic effect observed in PCOS involves macrophages [32]. Thus, we then investigated the regulatory effect of KOK on mRNA expression of the pro-inflammatory cytokines (IL-1β, IL-6, TNF-α), chemokines (IL-8, MCP-1), growth factors (EGF, TGF-β), and iNOS in the ovaries of each group 20 days after DHEA injection and KOK administration (Table 3). The ovarian mRNA levels of IL-1β (by 2.9-fold), IL-6 (by 3.2-fold), TNF-α (by 50.3-fold), IL-8 (by 2.7-fold), MCP-1 (by 6.0-fold), and iNOS (by 8.0-fold) were significantly elevated in the DHEA group compared to the mRNA levels in the normal group. However, the increased mRNA levels were decreased significantly by 52.6%, 58.2%, 86.9%, 57.9%, 75.3%, and 79.1%, respectively, in the DHEA + KOK group (Table 3). In contrast to these data, the mRNA expression of EGF and TGF-β was decreased by 36.8% and 60.9%, respectively, in the DHEA group compared to the mRNA levels in the normal group, and their decreased level was significantly up-regulated by 2.1-fold and 1.4-fold, respectively, in KOK administrated group (Table 3). KOK itself had no effect on the expression of these factors (Table 3). These data demonstrated KOK-mediated regulation of the expression of inflammatory mediators in ovary upon DHEA-induced PCOS model in rat.

**Table 3:** Effect of pre-administration of KOK on the expression of inflammatory mediators.

Gene	Normal (n = 6)	DHEA (n = 6)	DHEA+KOK (2.0 g/kg) (n = 6)	KOK (2.0 g/kg) (n = 6)
**IL-1β**	1.0±0.0	2.87±0.40^##^	1.36±0.08^**^	1.07±0.04
**IL-6**	1.0±0.0	3.18±0.43^##^	1.33±0.25^**^	0.98±0.07
**TNF-α**	1.0±0.0	50.3±24.1^##^	6.58±3.11^**^	0.95±0.07
**IL-8**	1.0±0.0	2.76±0.29^##^	1.17±0.15^**^	0.89±0.13
**MCP-1**	1.0±0.0	5.98±1.28^##^	1.48±0.19^**^	1.01±0.22
**EGF**	1.0±0.0	0.37±0.08^##^	0.79±0.05^**^	0.96±0.04
**TGF-β**	1.0±0.0	0.61±0.03^##^	0.83±0.08^**^	0.96±0.06
**iNOS**	1.0±0.0	7.95±1.08^##^	1.66±0.18^**^	1.11±0.15

Pre-administration of KOK diminished the increased mRNA expression of the pro-inflammatory cytokines (IL-1β, IL-6, TNF-α), chemokines (IL-8, MCP-1), and iNOS in the ovaries and increased the reduced mRNA expression of the growth factors (EGF, TGF-β) by DHEA injection. Values represent the mean ± SEM. ^##^p<0.01 normal vs. DHEA group. **p<0.01 DHEA vs. DHEA + KOK group. ANOVA with a Fisher's post-hoc test.

### Effect of KOK post-administration in DHEA-induces PCOS

**Figure 7:**
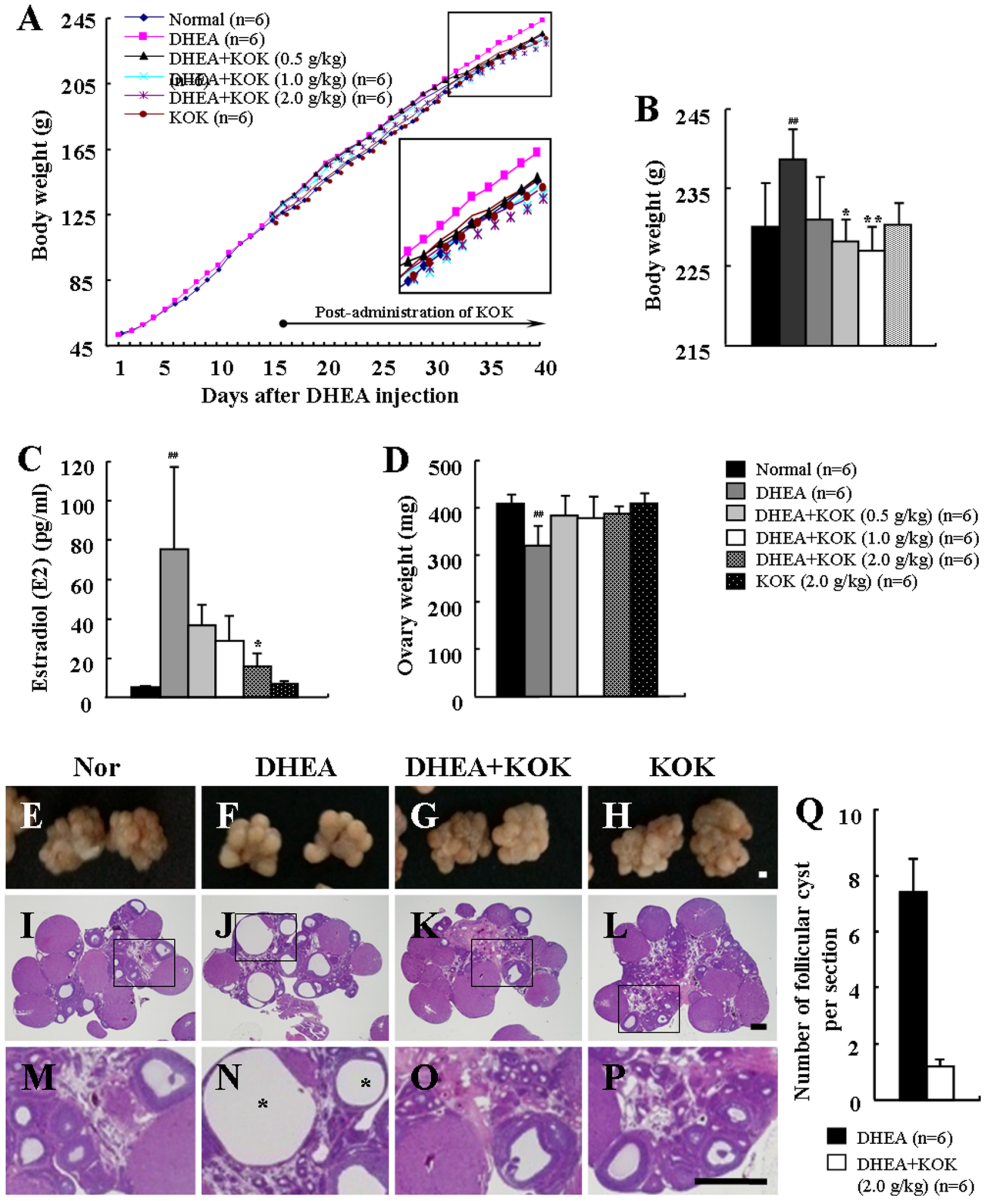
Effect of post-administration of KOK in body weight and ovarian morphology in in rats with PCOS. (**A**) Growth curves for the period of the treatment for normal, DHEA, DHEA + KOK, and KOK groups. Values represent the mean. (**B–D**) The graphs for body weight (B), serum estradiol (C), and ovary weight 40 days after DHEA injection (D). Values represent the mean ± SEM. ^##^p<0.01 normal vs. DHEA group. *p<0.05, **p<0.01 DHEA vs. DHEA + KOK group. ANOVA with a Fisher's post-hoc test. (**E–P**) Photos and photomicrographs of ovaries from normal (E, I, and M), DHEA (F, J, and N), DHEA + KOK (G, K, and O) and KOK group (H, L, and P). The rectangles indicate the area magnified in photos shown in panels M-P. (**Q**) Number of follicular cysts. Post-administration of KOK restored the follicular cysts (asterisks) induced by DHEA. Scale bar  = 50 μm.

**Figure 8:**
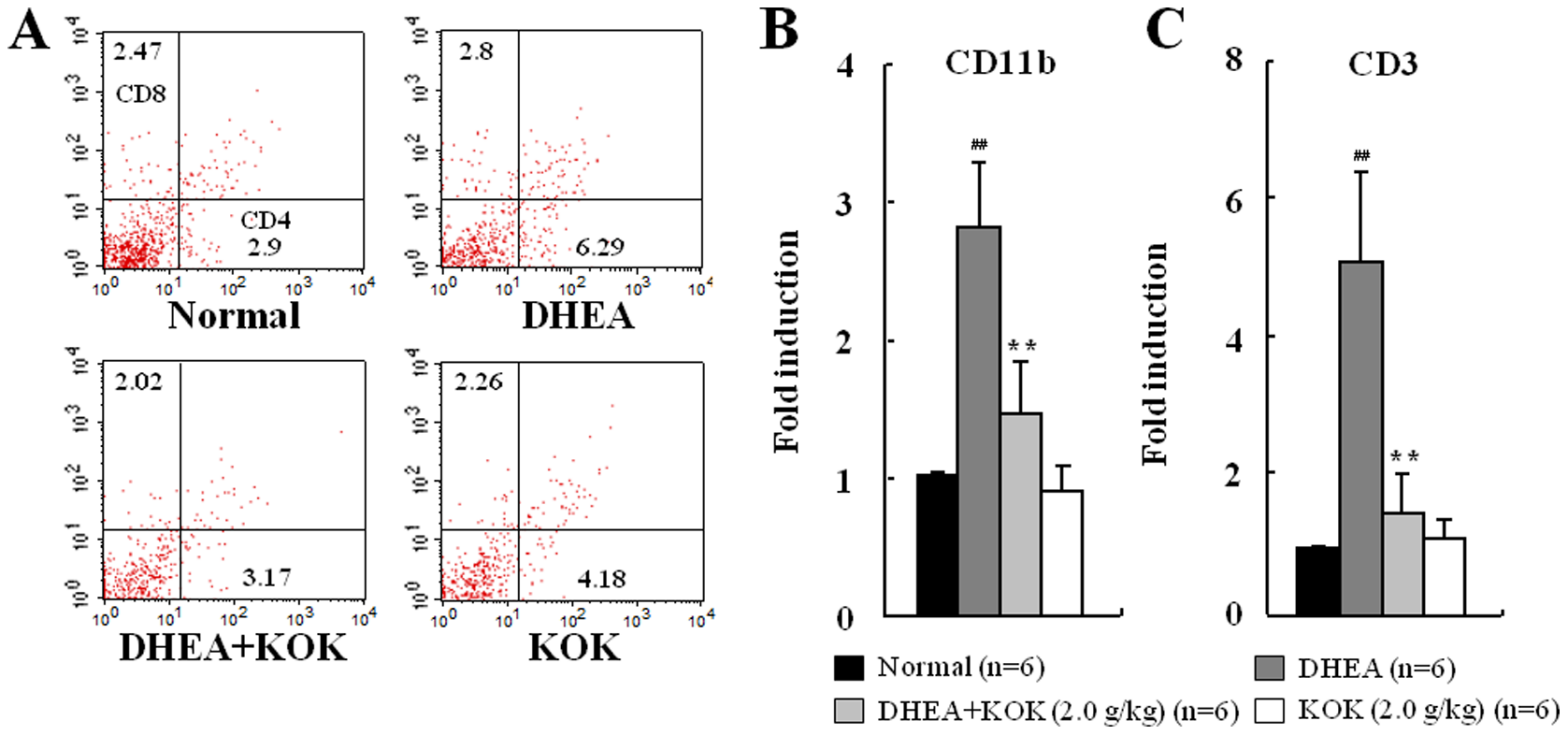
Effect of KOK post-administration in T lymphocyte expression in lymph nodes and macrophage infiltration to ovarian tissue. (A) Frequency of CD4 (+) and CD8 (+) cells in lymph nodes. Representative dot plots from flow cytometric analysis of CD4 (+) and CD8 (+) are displayed. Values represent the mean ± SEM. ^#^p<0.01 normal vs. DHEA group. *p<0.01 DHEA vs. DHEA + KOK group. ANOVA with a Fisher's post-hoc test. (B and C) Ovarian mRNA expression of CD11b and CD3. Post-administration of KOK inhibited the elevated mRNA expression of CD11b and CD3 by DHEA. Values represent the mean ± SEM. ^##^p<0.01 normal vs. DHEA group. **p<0.01 DHEA vs. DHEA + KOK group, ANOVA with a Fisher's post-hoc test.

As above, we confirmed that pre-administration of KOK improved DHEA-induced PCOS-like symptoms, which could suggest a preventive effect of KOK in PCOS. In addition, we investigated whether post-administration (administration after induction of PCOS) of KOK could restore the DHEA-induced PCOS-like symptoms in rat. The results were generally similar to those of pre-administration of KOK in PCOS model (Figs. 7 and 8, Table 4). Post-administration of KOK inhibited the increased body weight (by 4.8%), increased serum levels of estradiol (by 61.6%), increased number (by 84.6%) and size of follicular cysts, increased mRNA levels of IL-1β (by 51.8%), IL-6 (by 55.8%), TNF-α (by 74.8%), MCP-1 (by 62.5%), and iNOS (by 76.6%), increased population of rats with estrus cycle arrest compared with DHEA group. However, the mRNA expression of EGF and TGF-β was decreased by 70.7% and 60.2%, respectively, in the DHEA group compared to the mRNA levels in the normal group, and their decreased level was significantly up-regulated by 2.1-fold in KOK post-administrated group (Table 4). Generally, there were some differences compared to pre-administration. In the case of DHEA injection for 40 days, ovary weight was significantly less compared to the normal group (Fig. 7D). Also, the number of follicular cyst in the ovaries in the DHEA+KOK group (DHEA group, 7.8±1.3; DHEA+KOK group, 1.2±0.6) was decreased more than that of rats pre-administered KOK (Figs. 7E-7Q). Post-administration of KOK inhibited by 49.7% the increased activation of CD4 (+) T lymphocytes (by 2.2-fold) in lymph nodes by DHEA (Fig. 8A). Post-administration of KOK also inhibited the increased mRNA expression of CD11b (macrophage marker) and CD3 (T lymphocyte marker) in lymph nodes by DHEA (Fig. 8B and 8C). The results suggested that post-administration of KOK can improve the PCOS by DHEA injection.

**Table 4:** Effect of post-administration of KOK on the expression of inflammatory mediators in ovarian tissue.

Gene	Normal (n = 6)	DHEA (n = 6)	DHEA+KOK (2.0 g/kg) (n = 6)	KOK (2.0 g/kg) (n = 6)
**IL-1β**	1.0±0.0	3.77±0.55^##^	1.81±0.19^**^	0.98±0.06
**IL-6**	1.0±0.0	2.99±0.24^##^	1.32±0.12^**^	1.01±0.06
**TNF-α**	1.0±0.0	7.08±0.75^##^	1.79±0.14^**^	0.99±0.08
**MCP-1**	1.0±0.0	5.45±0.46^##^	2.05±0.24^**^	1.3±0.14
**EGF**	1.0±0.0	0.29±0.04^##^	0.62±0.07^**^	1.0±0.1
**TGF-β**	1.0±0.0	0.4±0.04^##^	0.84±0.09^**^	1.02±0.06
**iNOS**	1.0±0.0	7.38±0.39^##^	1.73±0.23^**^	1.16±0.09

Post-administration of KOK diminished the increased mRNA expression of the pro-inflammatory cytokines (IL-1β, IL-6, TNF-α), chemokines (MCP-1), and iNOS in the ovaries and increased the reduced mRNA expression of the growth factors (EGF, TGF-β) by DHEA injection. Values represent the mean ± SEM. ^##^p<0.01 normal vs. DHEA group. **p<0.01 DHEA vs. DHEA + KOK group. ANOVA with a Fisher's post-hoc test.

### Administration of KOK does not induce toxicity

Finally, to examine whether the administration of KOK induces toxicity to rats, we treated normal female, 23-day-old, prepuberal rats with 2.0 and 4.0 g/kg/day of KOK for 22 days and assessed toxicity in blood, liver, and kidney (Figure S1). No evidence of a toxic effect was evident, compared to those of normal rats without any saline or sesame oil treatment. Mean body weight (Figure S1A) and mean serum level of the most often used indicators of liver damage or disease - AST, ALT, and LDH (Figure S1B) were not affected by the prolonged administration of KOK. Also, histological structure (Figure S1C), distribution of apoptotic cells (Figure S1D), expression of caspase-3 (Figure S1E), and mRNA expression of the most often indicators of liver damage or disease - TNF-α and FAS in liver and kidney were not affected by the administration of KOK (Figure S1E). The results indicated that prolonged administration of 2.0 and 4.0 g/kg/day KOK does not induce toxicity.

## Discussion

Since PCOS is associated with metabolic disturbances and chronic inflammation, it has a significant impact on quality of life and has long-term health risks for affected individuals [34]. However, therapeutic strategies have not been evaluated. KOK is a well-known herbal prescription for patients with age-associated disorders in traditional Oriental medicine [13]. However, the efficacy of KOK has not been systematically analyzed until recently, and the efficacy determination relied on empirical results. Moreover, the mechanism underlying the effects of KOK on the female infertility has not been investigated. The present study was undertaken to identify the preventive and therapeutic potential of KOK on PCOS. We provide the first evidence that KOK has preventive and therapeutic potentials for PCOS.

The DHEA-induced PCOS model reproduces the main aspects of human PCOS, although its mechanism remains unknown [26], [30], [31], [35]. Our DHEA-induced PCOS model also modified the weight of the animals and ovaries, and the levels of serum glucose and estradiol agree with human PCOS. Therefore, we could infer that our PCOS model is suitable to test the effect of KOK. The body weight, adiposity, and adipocyte size are increased in animals and women with PCOS [36]. However, exercise reduces these factors corresponding with improved of PCOS in rats [36], [37]. Presently we confirmed the previous finding that KOK significantly reduces the increased body weight in rats with PCOS [36]. The result indicates the possibility that KOK can inhibit increased body weight by DHEA injection. DHEA-induced androgenisation induces the formation of follicular cysts in mouse ovaries, which appears at a higher frequency when treatment of DHEA is started at a younger age of 23 days and is continued for 20 consecutive days (until 43-days-of-age) [26]. Puberty (estrous cycle) of rat and human starts at 35–40-days-of-age [38] and 12–14 years, respectively, after birth. Therefore, DHEA-induced PCOS could be associated with the early onset (age ∼15 years) of PCOS in humans [39].

Hyperinsulinemia, a condition in which there is an excess level of insulin circulating in the blood, in women with PCOS leads to anovulation, impairs folliculogenesis, and affects follicular development [40]. Hyperinsulinemia is associated with insulin resistance in PCOS. In this study, although we did not directly measure insulin sensitivity, we assessed surrogate markers of insulin sensitivity such as fasting serum insulin, fasting blood glucose, and the HOMA index, which reflect the glucose-insulin relationship. The elevated level of glucose was reduced by pre-treatment of KOK, while the level of insulin and HOMA index was not affected. The results indicate that administration of KOK is not associated with insulin sensitivity in the DHEA-induced PCOS model.

The level of serum estradiol is increased along with formation of follicular cysts, estrous cycle arrest, altered ovarian steroidogenesis, and anovulation as a result of hyperandrogenism in PCOS patients [10] and in DHEA-/testosterone-induced animal models [30]. Recently, it was demonstrated that metformin restores ovulation in PCOS [41] and improves ovarian-related parameters in DHEA androgenised mice [31]. Consistent with these reports, we confirmed that KOK decreases the size and number of follicular cysts, restores estrous cycle arrest, and decreases the elevated serum estradiol in rats with PCOS. These results suggest that KOK could have potential activity in the treatment for PCOS. In the future, we should clarify how KOK treatment improves PCOS-related inflammatory symptoms.

Endocrine disturbances could be directly related to T lymphocyte differentiation and maturation. For example, estradiol and progesterone levels regulate the maturation of thymocytes, which is associated to autoimmune diseases, such as autoimmune premature ovarian failure [42]. CD8 (+) T lymphocytes was increased in premature ovarian failure patients [43] and in lymph nodes/ovarian tissue from DHEA-induced PCOS rats [44]. In the present study, the pre-administration of KOK reduced the increased percentage of CD8 (+) T lymphocytes in lymph nodes and the increased infiltration of CD8 (+) T lymphocytes in ovarian tissue with PCOS. The results indicate that pre-administration KOK could reduce the development of DHEA-induced PCOS by inhibiting the population and infiltration of CD8 (+) T lymphocytes. The estrogen receptor alpha is a novel pathological marker expressed by follicular dendritic cells in lymph nodes [45] and the estrogen deficiency following ovariectomy or menopause alters differentiation of T lymphocytes [46]. Serum estradiol also regulates T lymphocyte differentiation [42], [43], [47]. Presently, pre-administration of KOK down-regulated increased serum estradiol levels by DHEA treatment (Table 2). The result suggests that KOK could act in modulating the differentiation of T cells by inhibiting expression of estradiol, and indicate the important role of KOK in the regulation of some aspects of the complicated network that associate with the hormonal and the immunological pathway in a microenvironment of PCOS.

PCOS has been linked to chronic inflammation [33] and its major pathogenesis is macrophage contributions [32]. Ovarian macrophages product cytokines, chemokines, and growth factors in normal, inflammatory, and disease processes of ovary. The macrophages can orchestrate tissue remodeling and apoptosis, both of which are involved in folliculogenesis, ovulation, and corpus luteum formation [32]. Presently, pre-administration of KOK diminished the increased frequency of Iba-1 (+) macrophages in the theca cell layer of cysts and stroma, along with increased mRNA expression of CD11b and CD3 in ovarian tissue with PCOS (Fig. 6). The results suggest that KOK has an anti-inflammatory action in the DHEA-induced PCOS model. Given that macrophages have an important action in PCOS patients [32], numerous studies have compared serum and follicular fluid cytokine levels in PCOS patients. Serum and follicular fluid TNF-α and IL-6 levels are elevated in non-obese/non-diabetic PCOS patients treated with gonadotrophins [48] and MCP-1 and macrophage inflammatory protein-1 alpha are increased in PCOS patients and associated with adiposity [49]. Activity of TGF-β, iNOS, and cyclooxygenase-2 (COX-2) are increased in ovaries of PCOS patients [35], [50], and activity of iNOS and COX-2 can be prevented by metformin administration [35]. These results suggest that the immune system is involved in the pathogenesis of PCOS. We presently confirmed that pre-administration of KOK significantly decreased the increased mRNA levels of IL-1β, IL-6, TNF-α, IL-8, MCP-1, and iNOS in ovaries with PCOS. KOK increased the decreased mRNA expression of EGF and TGF-β in ovaries with PCOS (Table 3). These data demonstrate that KOK regulates the expression of inflammatory mediators in ovary PCOS. Targeting this inflammatory process by means of anti-inflammatory agents might be a therapeutic alternative to the current treatment.

In addition to the preventive effect by pre-administration of KOK, we investigated therapeutic potential by post-administration of KOK for DHEA-induced PCOS. The results of post-administration were generally similar to those of pre-administration of KOK in PCOS model (Figs. 7 and 8, Table 4). Post-administration of KOK further decreased the increasing of body weight by DHEA, more conclusively reduced the number of follicular cyst in the ovaries, and inhibited the activation of CD4 (+) T lymphocytes in lymph nodes than that of pre-administration of KOK (Fig. 7). The results suggest that post-administration of KOK can restore DHEA-induced PCOS. In the future, we should investigate the subtle difference of pre- and post-administration of KOK in PCOS-related inflammatory symptoms in DHEA-treated rats.

Major herbs consisting of KOK are among the well-known traditional East-Asian medicinal herbs [13]. *Rehmannia glutinosa* Liboschitz has been traditionally prescribed to reduce fever, regulate immunity, and improve various diabetic disorders [51], [52]. *Lycium chinense* Miller is used as a remedy for chronic liver and kidney disorders in Asia [13]. It improves trimethyltin-induced learning and memory deficits in the rats [53] and prevents or alleviates oxidative stress-induced hepatotoxicity [54]. *Aquillaria agallocha* Roxburgh contains 4-butyl-α-agarofuran as a major ingredients and show significant anti-anxiety activity in animal models [55]. *Poria cocos* Wolf is a fungus that grows around the roots of pine trees in East-Asian and North America [56]. Its inner parts are used to treat chronic gastritis, acute gastroenteric catarrh, gastric atony, edema, nephrosis, dizziness, nausea, and emesis [56], [57]. Its chemical constituents mainly include triterpenes, polysaccharides, and steroids [56], [58]. *Panax ginseng* C.A. Meyer is a perennial herb of the family *Araliaceae* containing ginsenosides as major active ingredients [59], [60]. *Panax ginseng* or ginsenosides increase physical strength, prevent aging, increase vigor [59], and produce immune, endocrine, cardiovascular, nervous, and cancer-related benefits [60]. Although it remains unknown which herb in KOK confers the beneficial effect for PCOS, the present data is the first step towards this identification.

## Conclusions

We investigated the protective effect of KOK in a DHEA-induced PCOS rat model. Pre-administration of KOK significantly decreased the elevated weight of body and ovary, the elevated size and number of follicular cysts, the elevated level of serum glucose and estradiol after the DHEA injection. KOK also reduced the elevated percentage of CD8 (+) T lymphocytes in lymph nodes, the elevated mRNA expression of CD11b and CD3 in ovaries, and the infiltration of macrophages in ovarian tissue with PCOS. KOK diminished the increased mRNA expression of the IL-1β, IL-6, TNF-α, IL-8, MCP-1, and iNOS in the ovaries and increased the reduced mRNA expression of the EGF and TGF-β. Interestingly, the post-administration of KOK also improved the DHEA-induced PCOS, generally similarly to those of pre-administration of KOK. These multiple effects of KOK may synergistically prevent and improve DHEA-induced PCOS via anti-inflammatory action, indicating its preventive and therapeutic potential for suppressing PCOS.

## Supporting Information

Figure S1 Administration of KOK does not induce toxicity. (**A–F**) To examine whether the administration of KOK induces toxicity to rats, normal female, 23-day-old, prepuberal rats were treated with 2.0 and 4.0 g/kg/day of KOK for 22 days and toxicity was assessed in blood, liver, and kidney. No evidence of a toxic effect was evident, compared to those of normal rats without any saline or sesame oil treatment. Mean body weight (A) and mean serum level of AST, ALT, and LDH (B) were not affected by the administration of KOK for long-term. Also, histological structure (C), population and expression of apoptotic cells and caspase-3 (D and E), and mRNA expression of TNF-α and FAS (F) in liver and kidney with KOK administration were not significantly affected by the administration of KOK. Arrows, TUNEL- or Caspase-3-positive cells.
(TIF)

Materials and Methods S1 Instrumentation and liquid chromatography-tandem mass spectrometry (LC-MS/MS) conditions.
(DOC)
