# Supplementary figures and images for: Oriental Medicine Kyung-Ok-Ko Prevents and Alleviates Dehydroepiandrosterone-Induced Polycystic Ovarian Syndrome in Rats
Source: PLoS One. 2014 Feb 10;9(2):e87623. doi: 10.1371/journal.pone.0087623 (PMC3919730; doi:10.1371/journal.pone.0087623)

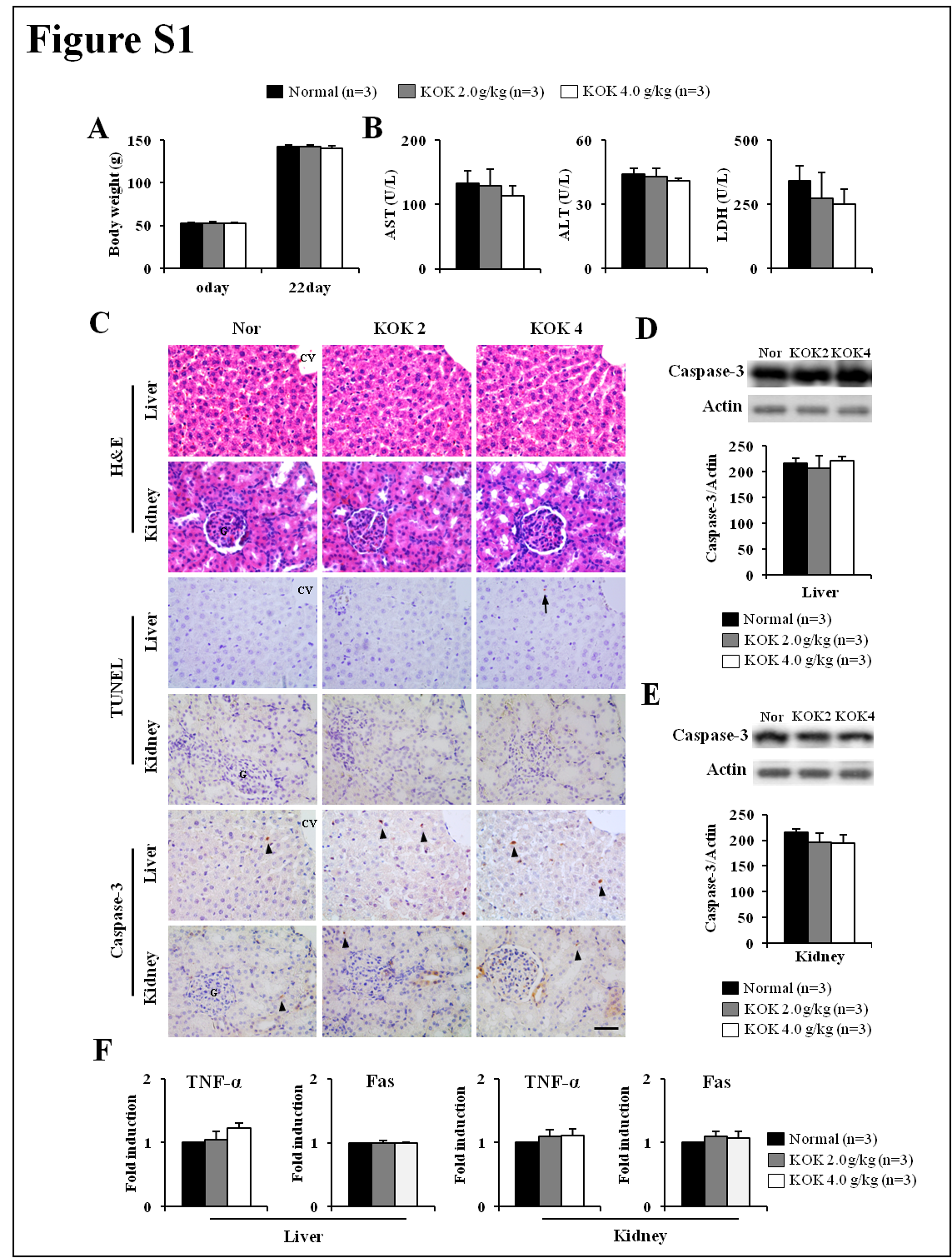

Supplement: Figure S1 — Administration of KOK does not induce toxicity. (A–F) To examine whether the administration of KOK induces toxicity to rats, normal female, 23-day-old, prepuberal rats were treated with 2.0 and 4.0 g/kg/day of KOK for 22 days and toxicity was assessed in blood, liver, and kidney. No evidence of a toxic effect was evident, compared to those of normal rats without any saline or sesame oil treatment. Mean body weight (A) and mean serum level of AST, ALT, and LDH (B) were not affected by the administration of KOK for long-term. Also, histological structure (C), population and expression of apoptotic cells and caspase-3 (D and E), and mRNA expression of TNF-α and FAS (F) in liver and kidney with KOK administration were not significantly affected by the administration of KOK. Arrows, TUNEL- or Caspase-3-positive cells. (TIF) [file pone.0087623.s001.tif]
