# Supplementary material for: Oriental Medicine Kyung-Ok-Ko Prevents and Alleviates Dehydroepiandrosterone-Induced Polycystic Ovarian Syndrome in Rats
Source: PLoS One. 2014 Feb 10;9(2):e87623. doi: 10.1371/journal.pone.0087623 (PMC3919730; doi:10.1371/journal.pone.0087623)
Supplement: Materials and Methods S1 — Instrumentation and liquid chromatography-tandem mass spectrometry (LC-MS/MS) conditions. (DOC) [file pone.0087623.s002.doc]

**Materials and Methods S1. Instrumentation and liquid chromatography-tandem mass spectrometry (LC-MS/MS) conditions**

LC-MS/MS analysis was performed with a Waters 2795 HPLC system and a Waters Micromass Quattro Premier triple quadrupole mass spectrometer equipped with a turbo electrospray interface in positive ionization mode (Waters Ltd., Watford, UK). The KOK sample was sealed in sterile bottles and retained at 4°C until used. The samples were filtered through 0.22 µm syringe filter and injected with 10 µL for LC-MS/MS analysis. Stock solutions (1000 μg/mL) of betaine and 5-hydroxymethylfurfural (5-HMF) were prepared in 100% HPLC grade methanol (J.T. Baker, Philipsburg, NJ, USA) to obtain working standards for calibration purposes. Calibration curves of betaine and 5-HMF were obtained at 1, 5, 10, 50, and 100 ng/mL and 0.1, 0.5, 1.0, 5.0, 10.0, µg/mL. Betaine and 5-HMF were detected by the multiple reaction monitoring scan mode with two channels of positive ion detection. The most abundant product ions of compounds were obtained at *m/z* 58.07 from the parent *m/z* 117.51 ion of betaine and at *m/z* 108.84 from the *m/z* 126.70 ion of 5-HMF. Data <was acquisited using Micromass Masslynx 4.0 and data processing was conducted using a Quanlynx data analysis program. A Capcellpak UG120 C_18_ (50×2.0 mm i.d. 3 μm; Shiseido, Kyoto, Japan) was used as analytical column and the mobile phase (consisted of 60% methanol and 40% Milli-Q water containing 0.1% acetic acid) was filtered through a 0.22 μm filter to degas before use, and the samples were analyzed at flow rate of 0.2 mL/min, 4°C of the autosampler, and 45°C of column oven. All other materials were of the highest grade commercially available.
